# Editorial: Community series in BCR signaling and B cell activation, volume 2

**DOI:** 10.3389/fimmu.2025.1533646

**Published:** 2025-01-15

**Authors:** Wenxia Song, Tae Jin Kim, Wanli Liu

**Affiliations:** ^1^ Department of Cell Biology and Molecular Genetics, University of Maryland, College Park, MD, United States; ^2^ Department of Immunology, Sungkyunkwan University School of Medicine, Suwon, Republic of Korea; ^3^ Personalized Cancer Immunotherapy Research Center, Sungkyunkwan University School of Medicine, Suwon, Republic of Korea; ^4^ State Key Laboratory of Membrane Biology, School of Life Sciences, Tsinghua-Peking Center for Life Sciences, Institute for Immunology, China Ministry of Education Key Laboratory of Protein Sciences, Beijing, China; ^5^ The First Affiliated Hospital of Anhui Medical University and Institute of Clinical Immunology, Anhui Medical University, Hefei, China

**Keywords:** B cell, B cell receptor (BCR), B cell activation, antibody, antigen, co-receptor, cytoskeleton

B cell receptor (BCR) signaling plays a fundamental role in B cell development, activation, and antibody responses. The BCR consists of a membrane immunoglobulin (mIg), the antigen recognition subunit, and CD79a and CD79b heterodimer, the signal transducing subunit (1). It belongs to a family of receptors that do not contain intrinsic kinases and transduces signals by interacting with other signaling proteins and organizing supramolecular complexes. While BCR activation mechanisms have been studied extensively, many aspects remain controversial. With the development of new cellular and molecular tools, different models have been proposed. Accumulating evidence points to spatial reorganization of surface BCRs upon antigen binding. In the cross-linking model (CLM), BCR complexes in the resting state reside as monomers. When encountering multivalent antigens but not monovalent antigens, BCR monomers are cross-linked to initiate BCR signaling cascades. It was observed since 1967 that the BCR could be activated only by multivalent antigens, such as the F(ab)_2_ fragment of anti-BCR antibody rather than its Fab fragment of the antibody, favoring the CLM (2). Direct experimental evidence of the CLM came from fluorescence resonance energy transfer (FRET) microscopy (3), which demonstrates BCR-BCR interactions in living cells.

Although CLM explains the mechanism of BCR triggering by multivalent antigens, CLM cannot explain B cell responses by small-sized soluble antigens. In the conformation-induced oligomerization model, the binding of membrane-bound monovalent antigens leads to surface BCR oligomerization, which is mediated by conformationally unmasked Fc interfaces (4). However, as the conformational unmasking requires some physical forces between epitope and paratope, this model cannot easily explain the BCR triggering by the soluble format of monovalent antigens (monovalent soluble antigen).

B cell response to monovalent soluble antigen may be better explained by the dissociation activation model. This model proposes that surface BCRs exist in an autoinhibitory oligomeric state in resting B cells, and BCR oligomers are dissociated upon antigen binding, which exposes phosphorylation sites of the Immunoreceptor tyrosine-based activation motifs (ITAMs) in the intracellular domain of CD79a/CD79b heterodimer and thus activates B cells (5).

Recently, the conformational change model has been proposed to explain how the signal from the extracellular domain interacting with different formats of external antigens can be transduced to the BCR intracellular portion (6). Here, the antigen-induced conformational changes of mIg could be transduced into conformational changes in the spatial relationship between CD79a and CD79b without the pre-requisition of BCR oligomerization. The conformational change of Fc region by antigen binding was also shown in secretory IgE (7). The reorientation of IgE Cϵ2 domains and intricate binding of FcϵRI to IgE Fc region introduced conformational changes and asymmetrical folding back of an IgE Cϵ2 domain on Cϵ4 and Cϵ3, explaining persistent activation of mast cells by IgE binding to allergen.

The functional status of the BCR is controlled and regulated by self-aggregation and its interaction with various partners in the membrane, such as the transmembrane phosphatase CD45 and the stimulatory coreceptor CD19. However, the structural basis for such dynamic interactions remains a challenging question. Recently published cryo-electron microscopic studies confirm the asymmetric organization of the BCR with one CD79a/CD79b heterodimer associating with one mIg and revealed structural interfaces between mIg, CD79a, and CD79b in the membrane and the extracellular space (8–10). Through analyzing the amino acid sequences and three-dimensional structural folding of the transmembrane domains of mIg, CD79a, CD79b, and their interacting partners, as well as other receptors and receptor complexes, in this Research Topic, Reth identified evolutionary conserved leucine zipper motifs in their transmembrane α-helixes. He further proposed potential functions for such motifs in mediating the interactions between mIg and the CD79a/CD79b heterodimer within the BCR and between the BCR and its transmembrane interacting partners, facilitating the formation of different immunoreceptor organizations and signal complexes. Therefore, this motif was named as an immunoreceptor organization and coupling motif (ICOM). While the function of the ICOM motifs remains to be tested, the proposed hypothesis is highly intriguing.

One of the earliest signal events in BCR activation is Ca^2+^ flux, which is critical for B cell development, proliferation, and differentiation (12, 13). Ca^2+^ flux is triggered by BCR-induced activation of phospholipase Cγ2, which generates inositol 1,4,5-triphosphate (IP_3_). IP_3_ activates the IP_3_ receptor on the endoplasmic reticulum (ER), leading to Ca^2+^ release from the ER. Decreased ER Ca^2+^ levels activate stromal interaction molecule (STIM) proteins on the ER membrane. Active STIM binds to and opens calcium release-activated calcium (CRAC) channels on the plasma membrane, leading to Ca^2+^ influx (11, 12). How BCR-induced Ca^2+^ influx is regulated and the roles of Ca^2+^ influx in different activation, differential, and functional stages of B cells remain elusive. In this Research Topic, Mahtani et al. investigated the role of a member of the calcium-permeable transient receptor potential ion channel family, TRPV5, in B cells using a TRPV5 knockout mouse model. Even though TRPV5 knockout did not significantly impact Ca^2+^ influx induced by soluble antigen and B cell development, it did cause impaired B cell spreading and contraction in response to membrane-bound antigens and reduced BCR signaling and early T-dependent antigen-specific responses. Also, in this Research Topic, Riobo and Yuseff examined the remodeling of the ER, an intracellular Ca^2+^ storage, during the immune synapse formation and found that the ER was redistributed towards the immune synapses and around the microtubule organization center in response to membrane-bound antigens. Such ER reorganization was regulated by the stiffness of the antigen-presenting surface. However, the relationship between this antigen-triggered ER reorganization and Ca^2+^ signal regulation has yet to be examined.

BCR signaling regulation is essential to maintain the balance of appropriate B cell activation toward non-self-antigens but not self-antigens. Although the antigen-binding affinity of the BCR is generally translated into the strength of BCR signaling, the fine-tuning of BCR signaling is required to generate effective antibody responses and prevent autoreactive responses. The BCR signaling is finely tuned by the interaction of the BCR with signaling molecules or costimulatory molecules. In this Research Topic, Hong and Kwak discussed specific traits of antigens with pathogenic potential and their impacts on BCR signaling and B cells’ ability to distinguish between self and non-self-antigens. The physical properties of antigens, such as solubility or stiffness and antigen spacing or valency, can regulate the organization of the BCR signalosomes or dynamic interactions among the signaling molecules. Proximal BCR signaling is initially triggered via tyrosine phosphorylation of ITAMs in the cytoplasmic tails of CD79a and CD79b by the Src family or Syk tyrosine kinases. While the role of tyrosine kinases in BCR signaling is well known, the role of serine/threonine kinase (STK) in BCR signaling is not completely understood. Han et al. discussed the role of various STKs in B cell development and antibody function.

The tonic BCR signaling that is independent of antigenic engagement is essential for B cell development and survival, but its establishment and maintenance remain elusive. The constitutive activation of PI-3 kinase has been shown to rescue the B cell survival signaling in BCR-deficient B cells (13). Research Topic, Pérez-Pérez et al. reported that Lrba-/- B cells showed heightened basal signaling, such as elevated NF-κB activation and Nur77 expression, but were defective in responding to BCR engagement, suggesting the involvement of LRBA in basal or tonic BCR signaling. In this Research Topic, Rossmanith et al. investigated the B cell defects in patients with ERCC2 mutation that is associated with trichothiodystrophy-1 (TTD1) and nucleotide excision repair. Patients with ERCC2 mutations exhibited hypogammaglobulinemia, decreased antibody responses upon vaccination, increased radiosensitivity, and decreased naive and transitional B cells, but the mechanism of defective BCR signaling in ERCC2-mutated B cells is still poorly understood.

This Research Topic brings together current understanding and recent advances in BCR signaling mechanisms, regulation, and their implications in B cell function and disease. The collected studies provide new insights into the complex molecular mechanisms underlying B cell activation and regulation, contributing to our understanding of immune responses and potential therapeutic targets.
